# The Use of PDX1 DNA Methylation to Distinguish Two Subtypes of Pancreatic Neuroendocrine Neoplasms with Different Prognoses

**DOI:** 10.3390/cancers15010160

**Published:** 2022-12-27

**Authors:** Hendrik Ungefroren, Björn Konukiewitz, Ulrich F. Wellner, Jörg Schrader, Tobias Keck

**Affiliations:** 1 First Department of Medicine, University Hospital Schleswig-Holstein (UKSH), Campus Lübeck, 23538 Lübeck, Germany; 2 Institute of Pathology, University Hospital Schleswig-Holstein (UKSH), Campus Kiel, 23538 Lübeck, Germany; 3 Department of Surgery, University Hospital Schleswig-Holstein (UKSH), Campus Lübeck, 23538 Lübeck, Germany; 4 First Department of Medicine, University Hospital Hamburg-Eppendorf, 20251 Hamburg, Germany

Pancreatic neuroendocrine neoplasms (pNENs) account for approximately 5% of all pancreatic tumors; thus, they constitute the second most common tumor type in the pancreas [1,2]. pNENs are classified into two groups according to the 2017 and 2019 WHO classifications of tumors of the digestive system [3]: well-differentiated pancreatic neuroendocrine tumors (WD-pNETs) and pancreatic neuroendocrine carcinoma (pNEC) [1,3]. WD-pNETs are further separated into three grading groups based on their proliferative indices and mitotic count. The WHO classifications have proven their significant value with respect to predicting the prognosis of pNENs, including in terms of tumor grade and differentiation. Most of the changes in the classification of pNENs occurred in 2017 when the molecular biological evidence had been consolidated, and the overall classification scheme was maintained in the 2019 WHO grading system [3,4]. The reported 5-year survival rates of G1, G2, and G3 tumors are 75%, 62%, and 7%, respectively [5]. The WHO classification represents a relatively simple guideline with excellent criteria that provide intuitive information regarding the overall prognosis of pNEN patients [4].

pNENs comprise a heterogeneous group of tumors, and risk stratification with which to predict the exact prognosis of the disease they cause remains a challenging task. Classical prognostic prediction is based on clinicopathological prognostic factors including—aside from the WHO grading system—the disease stage and serum biomarkers. One of the most reliable and practical general serum biomarkers is plasma chromogranin A. In functional pNENs, secreted hormones—such as insulin, glucagon, vasoactive intestinal polypeptide, gastrin, and somatostatin—are also useful for predicting recurrence and treatment responses, besides their utility as diagnostic tools [6]. Unfortunately, a ubiquitously applicable serum biomarker is currently unavailable, which is mainly due to the heterogenous nature of pNENs [7]. Serum biomarkers mainly include secretory products, which lack sufficient diagnostic sensitivity, specificity, and prediction power for prognosis [6,7], but are, nevertheless, clinically important due to their ability to provide non-invasive and repeatable measurements.

More recently, the genetic landscape of pNENs has been revealed due to advances in next-generation sequencing technology, and the knowledge regarding the molecular features of pNENs has been systematically advanced with multi-omics analyses, such as high-throughput transcriptomics, epigenomics, proteomics, metabolomics, and radiomics. The molecular subtyping of pNENs has established novel classifications based on common multigene mutations, the large-scale loss of heterozygosity, copy number variations (CNVs), and islet cell type-specific signatures. This has also led to the identification of novel prognostic factors, particularly genetic mutations, non-coding RNAs, epigenetic signatures, metabolites, and radiologic features. In parallel, remarkable progress has been made regarding multifactorial prediction models involving molecular biomarkers and multi-omics sequencing in order to improve clinical management and predict prognoses with a combination of factors rather than a single factor, for which the former appears to be more efficacious [8,9,10,11,12,13]. However, a comprehensive understanding of pNEN biology remains elusive [14], and the clinical, pathological, molecular, and epigenetic properties of pNENs still need to be further refined. Moreover, it is still difficult to apply the molecular profiling of pNENs in clinical routines for treatment prediction since a few targetable alterations associated with treatments have not yet been validated in clinical studies [15]. 

To provide but a few examples, comprehensive transcriptomic profiling has led to a novel histological classification of sporadic pNENs, which cluster into three subtypes: metastasis-like primary (MLP), islet/insulinoma (IT), and intermediate pNENs [16,17]. MLP-subtype pNENs preferentially express genes of pancreatic progenitors or immature islet cells with a higher frequency of *MEN1* and *DAXX*/*ATRX* mutations than in the other subtypes. The IT subtype exhibits features of mature β-cells with *INS* and *PDX1* expression, while intermediate pNENs show mature β-cell features with the expression of *GCG*, *NKX2-2*, and *GATA* genes. These subtypes also exhibit distinct metabolic profiles, thus suggesting that they arose through different tumorigenic pathways. Recently, another proteotranscriptomic-based classification of pNENs was introduced [18]. In the related study, metabolism-related molecular differences in an α-cell-like subgroup and the involvement of the Hippo signaling pathway in a stromal/mesenchymal subgroup were uncovered, and pNENs were subclassified into four groups: α-cell-like, stromal/mesenchymal, proliferative, and PDX1^high^. Mutant *MEN1*/*DAXX* and metabolic features characterize an α-cell-like subgroup, while elevated YAP1 and WWTR1 activity denotes the stromal/mesenchymal subgroup. The proliferative subgroup, exhibiting the molecular features of increased cell proliferation, consisted of roughly equal proportions of pNETs and pNEC, which strongly suggests that, at the molecular level, a certain subset of pNETs is more like pNEC than other pNETs. 

pNENs can be classified as functional or non-functional pNENs based on the presence or absence, respectively, of symptoms associated with the overproduction of specific hormones [19]. The production of these hormones is indicative of the cell of origin, with insulinomas (functional pNENs overproducing insulin) likely originating from pancreatic β-cells [20]. In contrast, non-functional pNENs are believed to originate from different cell types, which accounts for the observed heterogeneity in the clinical characteristics and prognoses in this patient group. Our knowledge regarding the cells of origin of non-functional pNENs remains incomplete [20]; however, their classification according to their cell origins is gaining more attention due to the recent development of multi-omics studies. 

There is still limited knowledge regarding the genome-wide DNA methylation changes precipitated by pNENs. More specifically, the exact changes in DNA methylation between pNENs and their presumed cells of origin, the endocrine cells of the pancreas, remain largely unknown. Recently, two subtypes of pNENs have been described that were linked to a cell of origin and have different prognoses. A difference in the expression of the transcription factor PDX1 was one of the key molecular differences. As mentioned above, *ATRX*, *DAXX*, and *MEN1* (A-D-M) are frequently mutated in pNENs, and mutations in these genes have been linked to prognosis [21]. Recently, two publications provided experimental evidence regarding a possible link between the cell of origin/cell identity, A-D-M mutations, DNA methylation, and the expression of specific genes, namely, *ARX* and *PDX1* [22,23,24,25]. Chan and coworkers compared the gene expression and DNA methylation profiles of tumors that are A-D-M-mutated or A-D-M wild type (WT) [22]. They observed that the somatic mutations of A-D-M occur exclusively in pNENs of the α-cell type and were associated with high ARX and low PDX1 gene expression, the hypermethylation of four CpGs in the *PDX1* promoter of the mutant tumors, and a worse prognosis than tumors with WT A-D-M [22]. In this study, the expression of *ARX* and *IRX2* was specific for α-cell-like pNENs, while *PDX1* expression was specific for their β-cell-like counterparts. This was consistent with the low DNA methylation levels in the *PDX1* promoter in β-cells and the high DNA methylation levels in the *PDX1* promoter in α-cells [24]. Chan and colleagues also found that mutated and WT tumors clustered separately based on *PDX1* expression and DNA methylation profiles, with mutant tumors having an expression profile similar to that of α-cells [22]. Another study suggested classifying pNENs according to the DNA methylation profiles of *IRX2*, *ARX*, and *PDX1*, which were divided into α-cell-like and β-cell-like subtypes [26]. These were further classified into different subtypes based on mutations of A-D-M and CNVs, while the mTOR and Hippo pathways were enriched in α-cell-like tumors. Furthermore, Cejas et al., performed subtyping of non-functional pNENs into two major subtypes, designated A and B, based on expression patterns and histone modifications in enhancer regions, which resemble α and β-cells, respectively [23]. Specifically, based on the H3K27 acetylation pattern, they identified super-enhancer regions containing genes that play an important role in defining cell identity [23,25]. The ARX (in subtype A pNENS) and PDX1 (in subtype B pNENs) regions fulfilled super-enhancer criteria, and, of note, distant relapses predominated in the patients with α-cell-like (ARX+) tumors. Interestingly, subtyping was also possible using immunohistochemistry for ARX and PDX1, and these subtypes were associated with a significant difference in relapse-free survival [23]. Yet another study revealed different classifications according to ARX, PDX1, INS and GCN expression data combined with tumor DNA methylation profiling to show that non-functional pNENs can be divided into three subtypes: α-cell-like, β-cell-like, and intermediate pNENs [27]. Intermediate tumors had a higher risk of relapse compared to α- and β-like tumors, and it was demonstrated that they harbor frequent A-D-M mutations and whole-chromosome losses. 

To explore this further, Boons et al. [28] have performed an exploratory genome-wide DNA methylation analysis on 26 pNENs and compared this to the methylation profiles of the pancreatic islets of five healthy donors. In addition, they evaluated the DNA methylation of *PDX1*, which has not yet been used for pNEN subtyping, even though quantitative changes in PDX1 expression constitute one of the most important distinguishing features between the recently described subtypes. To evaluate *PDX1* DNA methylation’s potential with respect to its performance in subtyping and as a prognostic marker, this group has further combined data from Infinium Methylation EPIC arrays, which investigate numerous CpGs, with data from additional Illumina 450 K arrays, thereby yielding an impressive cohort of 83 pNENs. In addition, the methylation profile of the *PDX1* region was used to subtype a global cohort of 83 pNEN, 2 healthy α-cell, and 3 healthy β-cell samples. In this study, 26,759 differentially methylated CpGs and 79 differentially methylated regions were identified. Although the number of healthy α- and β-cell samples could have been higher, the authors found that pNENs have a genome-wide DNA methylation profile that is different from that in normal pancreatic islets. In addition, they demonstrated that this difference can be used to distinguish two pNEN subtypes, A and B, and illustrated a link to their respective cells of origin, namely, α and β-cells. Moreover, this study validated, for the first time, that besides PDX1 expression, the use of the DNA methylation profile of the *PDX1* region can be used as an additional (or alternative) method for the subtyping of pNENs. Additionally, at the molecular level, the two subtypes appear to be different, with different CNA patterns and with A-D-M mutations more commonly observed in subtype A pNENs. Finally, gene set enrichment analysis has indicated several pathways that are affected by these DNA methylation alterations, including the ERK–MAPK pathway and immune system-related pathways. The authors speculate that the molecular drivers in these pNEN subtypes may differ, and that this could affect the choice of treatment regimens in clinical practice. Most importantly, these subtypes had different clinicopathological characteristics and a different prognosis, with subtype A pNENs having a significantly worse prognosis compared to subtype B pNENs. Moreover, they exhibited a different risk of relapse, which supports the use of a different strategy in clinical follow-ups, with a closer follow-up for subtype A patients.

The recently suggested classifications based on multi-omics features now enable a thorough classification of pNENs, which can be used to achieve a comprehensive understanding of the disease and to predict clinical outcomes more precisely. However, since a couple of differences exist among the various molecular factor-based-subtyping strategies, a more integrated classification method is needed in the future.
